# Cow-Assisted Interventions in Social Farming: First Results of a Pilot Study

**DOI:** 10.3390/ani15202957

**Published:** 2025-10-13

**Authors:** Biancamaria Torquati, Giulia Angelucci, Silvana Diverio

**Affiliations:** 1 Department of Agricultural Food and Environmental Sciences, University of Perugia, Borgo XX giugno, 74, 06121 Perugia, Italy; giulia.angelucci@unipg.it; 2 Laboratory of Ethology and Animal Welfare (LEBA), Department of Veterinary Medicine, University of Perugia, Via San Costanzo 4, 06126 Perugia, Italy; silvana.diverio@unipg.it

**Keywords:** animal-assisted interventions, social farming, cow, animal welfare, mental health disorders, eating disorders, adolescents removed from families, cost analysis

## Abstract

**Simple Summary:**

Cows are usually seen only as farm animals, yet they can also play a role in supporting people’s well-being. This study tested for the first time in Italy whether working with cows could help people facing personal difficulties. On a farm in Umbria, four cows took part in Animal-Assisted Interventions with three different groups of participants: teenagers living away from their families, young adults with mental health challenges, and people with eating disorders. Participants spent time feeding, brushing, leading, and observing the cows. These activities boosted self-confidence, encouraged emotional expression, and improved social interactions among participants. For those with eating disorders, the experience also reduced negative feelings toward dairy products. The cows remained calm and comfortable throughout, showing that the activities did not harm their welfare. The cost analysis highlighted the relative affordability of cow-assisted activities, with the main costs coming from the staff needed to organize and guide the assisted activities. Our findings suggest that cows can contribute to innovative forms of care and education, opening new opportunities for social farms to support people with disabilities.

**Abstract:**

Social farming combines agricultural, social, and healthcare functions, and Animal-Assisted Interventions (AAIs) are increasingly being applied within this framework. Despite their potential, cattle are excluded from Italian guidelines and rarely studied. This pilot study explored the feasibility, effects, and economic sustainability of cow-assisted interventions within social farming in Umbria, Italy. It represents an original and innovative contribution, drawing attention to the therapeutic potential of the human–cow relationship. The study presents an experimental cow therapy protocol and proposes behavioral monitoring tools designed both for people with different disabilities and for the animals involved. Four Red Pied Valdostana cows were involved in structured sessions with three groups: adolescents removed from families, young adults with mental health disorders, and individuals with eating disorders. Activities included observation, feeding, grooming, problem solving, and leading. Human outcomes were assessed regarding emotional, relational, and behavioral dimensions, and animal welfare was continuously monitored. A cost analysis was also conducted for Animal-Assisted Activity (AAA), Animal-Assisted Education (AAE), and Animal-Assisted Therapy (AAT). Participants reported improved self-esteem, emotional expression, and social interaction; the eating disorder group showed greater openness toward dairy consumption. Animal welfare remained stable with high tolerance to handling. Costs were driven mainly by professional staff rather than animal care, with average hourly costs of €74.51 (AAA), €144.99 (AAE), and €172.41 (AAT). The comparative analysis demonstrates a clear trade-off: as the intervention shifts from recreational (AAA) to educational (AAE) and finally to therapeutic (AAT), the financial investment increases in parallel with the level of professionalization, personalization, and expected clinical outcomes. Cow-assisted interventions proved to be safe, feasible, and beneficial, supporting their potential inclusion in Italian guidelines on AAIs.

## 1. Introduction

Social farming refers to the combination of agricultural activities with social, educational, and health-related services to benefit disadvantaged people and the wider community [1,2,3]. It represents one of the many ways in which farms can perform multiple functions beyond food production, in line with the broader concept of agricultural multifunctionality [4,5,6]. It is increasingly recognized not only as a productive activity but also as a tool for reinforcing the socio-economic fabric of rural areas by promoting social inclusion and local development. Historically, agriculture has always had a social dimension [7,8,9]. The life cycle of farming families and the close network of relationships within rural communities shaped the evolution of farms. Today, the intersection of agriculture and social engagement has acquired renewed significance [10,11,12].

On the one hand, social farming broadens the multifunctional scope of the profit-driven agricultural sector, allowing it to reclaim its social function—once fulfilled by traditional forms of mutual aid, solidarity, and assistance that characterized rural life [12,13]. On the other hand, it offers new opportunities for collaboration with non-profit organizations and the public sector. This synergy allows social agriculture to generate public goods related to community life and territorial identity, support the labor inclusion of marginalized individuals, and provide educational activities and cultural services connected to traditional, environmentally sustainable production processes and the preservation of biodiversity for families and institutions [3,5,14]. Territorial identity refers to the presence of socioeconomic context conditions allowing convergence between collective and private interests, and feeding a sense of belonging and loyalty to a community [15]. In this light, social farming should not be seen merely as a “virtuous practice,” but as a foundational element of a new model of rural development and participatory welfare, understood as a system in which communities, civil society organizations, and local institutions collaborate with public authorities in designing and delivering services [9,16].

Welfare systems today are increasingly challenged by population aging, migration, economic restructuring, social fragmentation, and resource constraints [17]. Social farming acts as a small-scale experimental laboratory in this context. It integrates agricultural production systems and rural development with the perspective of the social economy, understood as a socioeconomic movement that puts people and the planet before profits, relying on collective and democratically organized enterprises such as cooperatives, non-profit organizations, and mutual societies that engage in market activities with a social rather than profit-driven function [18,19], as well as social policy, and health services [5]. While agriculture expresses the needs of farms and territories, the social sector addresses the demands of families, social workers, and institutions. Their convergence fosters a “One Welfare” approach—an evolution of the One Health model—that emphasizes the interdependence of human, animal, and environmental well-being, and promotes social and relational sustainability [20]. In the wake of the COVID-19 pandemic, social farming has gained renewed relevance as a space where rural development goals intersect with social cohesion and community participation, aligning with the ambitions of the United Nations (UN) 2030 Agenda and the European Green Deal. European Union (EU) policies increasingly emphasize inclusive growth in rural areas and the need to develop innovative social services that also generate employment [4].

Italy stands out as the only EU country with legislation dedicated specifically to social farming: Law No. 141 of 18 August 2015. This law promotes social farming as a multifunctional practice that delivers social, healthcare, educational, and work integration services. It seeks to ensure equitable access to essential services, particularly in rural and disadvantaged areas. According to the law, social farming can be practiced by agricultural entrepreneurs (as defined in Article 2135 of the Civil Code), either individually or in association, and by social cooperatives whose revenues come primarily from agricultural activities. Among the four types of activities recognized by the law is one specifically involving “services that support and assist medical, psychological, and rehabilitation therapies, including the aid of farm animals and the cultivation of plants.” This provision establishes a legal and practical foundation for integrating Animal-Assisted Interventions (AAIs) within social farming. Although social farming and AAIs emerged from different traditions, they can be highly complementary, especially in rural areas. AAIs offer therapeutic and educational benefits that enrich social farming projects, while farms provide ideal settings for these interventions. This relationship is codified in Italian Ministerial Decree No. 12550 of 2018, which defines the operational standards of social farming in Italy.

The International Association of Human–Animal Interaction Organizations (IAHAIO) defines AAIs as structured, goal-oriented interventions in healthcare, education, and social services that incorporate animals for the purpose of therapeutic gains in humans [21,22]. These interventions address a wide range of populations, including individuals with physical, psychomotor, cognitive, or psychiatric conditions, as well as children and the elderly [23,24,25].

The Italian Guidelines on AAIs, published by the Ministry of Health in 2015, identify three forms of intervention: Animal-Assisted Therapy (AAT), Animal-Assisted Education (AAE), and Animal-Assisted Activities (AAA). These differ by their goals, target groups, and the professional teams involved. AAT and AAE, in particular, require multidisciplinary teams and a certified training path that includes preparatory, intermediate, and advanced courses. While AAIs in Italy traditionally involve companion animals such as dogs and cats—and occasionally horses, rabbits, and donkeys—there is little research on the role of farm animals, particularly cattle, in such programs. Notably lacking, are any studies on the cognitive effects of therapeutic work on farm animals themselves [26].

One emerging practice, cow cuddling, originated in the Netherlands and is based on the soothing presence of cows—their body temperature, mass, and calm demeanour are said to have a relaxing effect [27,28]. While it has gained popular attention, the scientific basis for such practices remains limited. Cows (members of the subfamily *Bovinae*) have coexisted with humans for approximately 10,000 years [29,30], yet their historic role has often been defined by utility rather than empathy—reflected in the etymological root of “cattle” as movable property [31]. Today, cows are still frequently viewed through an economic lens, which can obscure their emotional and cognitive capacities [32,33]. However, ethological research increasingly recognizes cows as intelligent, emotionally complex, and socially sophisticated animals [34,35]. Despite this, they remain underrepresented in structured AAI programs, often grouped generically as “farm animals,” with their therapeutic potential largely unexplored in isolation.

Recent literature reflects a growing but fragmented interest in cow-assisted interventions within the broader Green Care movement [36,37,38]. While cows may offer significant therapeutic potential, their roles remain poorly characterized, and their welfare is often underreported. Research to date is limited by small sample sizes, lack of standardized methods, and inconsistent outcome measures. A paradigm shift is needed to recognize cows not merely as passive participants, but as active therapeutic partners, whose well-being is integral to program success. This calls for species-specific protocols, ethical oversight, and rigorous, high-quality research.

The concept of multifunctional agriculture further supports the inclusion of cow-assisted activities as part of a diversified approach to farming, combining food production with social, educational, and environmental goals [39]. Some pilot studies also link cow-assisted activities with biodiversity preservation through the use of indigenous breeds [16,40]. The breeds used in such programs include Holstein [26], Norwegian Red [41], and Polish White-Backed [40]. It has been observed that Jerseys and Ayrshires have a nervous and reactive nature [42]. Jerseys, however, are smaller in size and therefore easier to handle than Friesian Holstein cows despite their temperament. Although early findings are encouraging, the field suffers from a lack of standardization and methodological rigor. Multiple reviews have called for consistent protocols and robust outcome measures [43,44,45]. Excessive regulation may, however, compromise the authenticity of AAI programs. A balanced approach is needed: one that preserves the organic nature of farm-based therapy while ensuring reproducibility and ethical integrity.

This pilot study, despite some limitations that are acknowledged in the paper, explored the feasibility, the behavioral effects on both human participants and cows, and the economic sustainability of cow-assisted interventions within social farming. It represents an original and innovative contribution, aiming to draw attention to the therapeutic potential of the human–cow relationship. Specifically, we present an experimental cow therapy protocol and propose behavioral monitoring tools designed both for people with different disabilities and for the animals involved. Moreover, the study addresses the economic dimension of animal-assisted interventions, considering both the direct and indirect management costs sustained by the social farm to deliver these activities, and the financial sustainability of the initial investment required to implement them.

## 2. Materials and Methods

### 2.1. Case Study

The pilot study was conducted at Forme dell’Anima (hereafter “the Cooperative”), a non-profit organization that has been developing innovative models of social farming for over a decade. The Cooperative is an organic, multifunctional farm where people live together in a community, sharing both work and housing, and it is guided by the principles of mutuality, solidarity, democracy, and strong territorial identity.

Its activities integrate (i) the traditional social role of farming, (ii) social agriculture initiatives consistent with national legislation, (iii) close interaction with urban and rural communities, and (iv) a strong emphasis on animal welfare and the human–animal relationship. Farming practices are based on biodynamics, synergistic agriculture, and permaculture, supporting environmental protection and high-quality agri-food chains. The Cooperative produces cereals, vegetables, legumes, dairy, and honey, and also promotes training, environmental education, and social tourism initiatives.

Cows, central to both production and community life, inspired the development of a pioneering form of AAI using bovines. Project activities were co-designed with stakeholders, with the Cooperative acting as a local development agent and innovator in welfare systems. Agricultural resources were reinterpreted within a multifunctional framework: dairy farming was seen as co-producing food (economic value), therapy and inclusion (social value), and environmental benefits (ecological value). This approach reflects the One Health and One Welfare perspectives, emphasizing the interconnectedness of human, animal, and environmental well-being.

The project, “Pilot and Evaluation of Cow Therapy Activities for the Well-being of Humans and Animals in Social Farming”, was implemented within the Umbria Rural Development Programme 2014–2020 (Measure 16.9). It established a network of public, private, and third-sector actors in the Narni-Amelia area (Umbria, Italy) to deliver innovative social services grounded in sustainable agricultural processes.

A multidisciplinary team was established at the start of the project, in accordance with national guidelines and after completing the three required training levels. The team included a veterinarian specialized in AAI, a psychotherapist acting as project coordinator, two psychologists serving as intervention coordinators, three animal co-handlers, and an agricultural economist. The project involved 4 Valdostana Red Pied cows from the small, well-managed herd of the Forme dell’Anima community farm. The cows involved in the project were accustomed to human presence and handling from birth, as they were born and raised on the farm. From their first days of life, they were caressed and bottle-fed by different people, including children, which fostered positive expectations toward humans. They were also regularly led outside the paddock and pasture, initially with a harness. This procedure was repeated frequently, and over time, the cows became so familiar with the routine that they would walk calmly beside the handler without requiring a harness.

As cattle are not included among the five species recognized by the national AAIs guidelines (dogs, cats, rabbits, horses, and donkeys), authorization for their use in AAT/AAE was requested from the National Reference Center and the Ministry of Health. The request was denied, mainly for safety reasons related to the animals’ size, although this justification appears somewhat inconsistent, considering that horses are also large animals and may present handling risks. Consequently, the project was limited to recreational AAA activities, in line with Ministry of Health Note No. 1373/2024, which allows the use of other domestic species for AAA without prior authorization.

In accordance with national AAI guidelines, the animals involved had been raised and habituated to human presence and interaction from an early stage. At Forme dell’Anima, cattle and calves were accustomed to handling, feeding (also by unfamiliar people), receiving care, playing, and being led with a halter inside and outside the paddock. Each animal was individually named from birth and generally responded to the call of its handler.

In line with national AAI guidelines, interventions were monitored with respect to both users and animals. Assessments were conducted at baseline, at the end of the project, and after each session, recording relational, emotional, and behavioral parameters, as well as user–animal interactions and care. Animals were monitored for emotional state and behavior during activities. A risk monitoring system was also implemented, which included informing participants of potential risks and behavioral rules, followed by an evaluation of their understanding. The service was developed by combining the expertise of a veterinarian specialized in AAIs with knowledge of the behavioral habits of the cattle raised at the cooperative. The initial design was refined through an experimental, trial-and-adjustment approach, taking into account the reactions of both users (e.g., limited engagement in some activities) and animals (e.g., preference for conspecifics or grazing over interaction), as well as factors related to the activity setting.

During the pilot, several adjustments were made regarding group size and session duration. It was found that groups of 4–6 participants were optimal, as larger groups created downtime for users and excessive workload for the cow. Sessions never exceeded one hour for the animal and often lasted less. Interactions included both individual (e.g., grooming) and group activities (e.g., problem-solving tasks). Interventions took place outdoors or in semi-open environments (paddock or stable), and were therefore scheduled mainly in mild seasons to ensure participant comfort and safety. Flexibility in session management was essential to adapt to the needs of both users (e.g., health conditions, mood changes) and animals (e.g., estrous cycle, herd-seeking behavior, resistance to handling).

### 2.2. The Beneficiaries

A total of 34 participants were involved in the project, and the AAIs were designed for three categories of beneficiaries (Table 1). The first group consisted of 8 adolescents removed from their families (aged 12–16 years; 5 males and 3 females). The second group included 12 young adults with mental health problems (aged 18–30 years; 8 males and 4 females). The third group comprised 14 individuals with eating disorders (aged 16–22 years; 2 males and 12 females).

The adolescents were sheltered at the “Bethel Community” in Amelia. These are children and adolescents who have been temporarily or permanently removed from their families due to situations of neglect, abuse, parental psychiatric disorders, substance addictions, or other conditions that endanger their well-being. The group also included unaccompanied foreign minors.

The young adults with mental health issues were recruited through the day center “Si Può Fare” in Narni, run by Intermunicipal Consortium for Social Services (CIPSS), which provides community-based programs for patients aged 18–30 under the care of local mental health services. The group consists mainly of individuals with personality disorders (bipolar, borderline) and, in some cases, a history of psychotic episodes or substance abuse, and meets twice weekly. Additional participants with similar conditions were included from the residential facility “Casa Avigliano”.

The third group consisted of young women with eating disorders, hosted at the therapeutic–rehabilitation community “Città Giardino” in Terni, managed by the Assistance and Solidarity Cooperative for Vulnerable Groups (Casaligha, in Italian). This semi-residential program provides intensive, multidisciplinary treatment combining psychotherapy, nutritional rehabilitation, assisted meals, and social skills development.

### 2.3. Experimental Operational Cow Therapy Protocol

The experimental cow therapy protocol consisted of 11 structured sessions, all conducted outdoors in the cattle area (preferably in the covered paddock) (Table 2). Activities combined educational, observational, and relational elements, progressively guiding participants from an introduction to the project and cattle behavior, to direct care and interaction (feeding, grooming, leading with a halter), creative and sensory experiences (art, music), and practical farm activities (cheese-making, milking). The protocol was designed to foster trust, empathy, collaboration, and awareness of animal welfare, while ensuring participant safety and comfort.

The sessions followed a progressive structure: initial meetings focused on understanding cattle behavior and safe interaction within the farm setting, beginning with observation through a fence, followed by feeding, and gradually moving to direct contact and more complex activities. This stepwise approach allowed participants to build confidence while ensuring respect for the animals’ pace.

Risk management was a core component of the protocol. At the first session, participants were informed about the animals’ characteristics and potential risks (size, weight, horns), and behavioral rules were reiterated throughout the program (e.g., avoiding loud noises, sudden movements, open footwear, food near animals, or leaning on fences). Compliance was consistently observed.

Handlers, who had strong, trusting relationships with the cattle, played a central role in guaranteeing safety for both animals and users. Cattle were always handled with a halter during sessions, and separate entry/exit routes for people and animals facilitated safe handling. First-aid equipment was available on-site, and all staff were trained in emergency procedures.

Potential sanitary risks were addressed through regular veterinary monitoring, biosecurity measures (disinfestation, disinfection, antiparasitic treatments), restricted animal contact outside the farm, and immediate diagnostic and therapeutic intervention in case of suspected disease. Hygiene and preventive rules were systematically applied, with appropriate training provided to both staff and participants.

The development of the experimental operational cow therapy protocol resulted in a structured pathway designed to safeguard the well-being of both humans and animals. The protocol is divided into ten core principles: (1) gradual progression of activities to allow participants and animals to adapt at their own pace; (2) a cycle of 10–12 sessions to build continuity in the human–animal relationship; (3) diversified activities addressing physical, emotional, and creative dimensions; (4) inclusive participation, with group size tailored to optimize benefits for both animals and people; (5) careful preparation of spaces to ensure a safe and functional setting; (6) continuous attention to animal welfare, including physical and emotional state; (7) adaptation of activities to weather and environmental conditions; (8) a balance of individual and group interactions; (9) operational flexibility to respond to emerging needs and animal reactions; and (10) systematic risk assessment and management to ensure safety.

### 2.4. Monitoring Tools

To systematically assess both beneficiaries’ progress and the welfare of the animals involved, a set of structured monitoring tools was developed and applied throughout the cow-assisted therapy sessions. These instruments were designed to capture different dimensions of the intervention, ranging from individual progress and emotional responses to nutritional attitudes and animal welfare.

Specifically, the monitoring protocol included five sheets as detailed in Table 3.

A structured battery of four observation sheets was developed to systematically monitor participants’ behavior, emotional states, and attitudes during the sessions. Observation sheet 1 focused on emotional expressions (happiness, disgust, anger, fear, avoidance) and relationships with others (active participation, participation only when asked, interaction, or withdrawal), rated on a four-point Likert scale (1 = never; 4 = always). Observation sheet 2 assessed interaction and care behaviors toward the cow, including eye contact, tactile contact, acceptance of approach, playing, brushing, feeding, petting, and interest in the animal’s condition, also rated on a four-point Likert scale. Observation sheet 3 monitored psychological symptomatology and self-esteem, including anxiety, phobias, depressed mood, obsessive behaviors, adequacy and insecurity, feelings of gratification and competence, passivity and indifference, and active or proactive behavior, again rated on a four-point Likert scale. Observation sheet 4 explored attitudes toward milk and dairy products, covering perceived nutrients (sugars, fats, proteins), taste appreciation, frequency of consumption, and variety of products consumed, with four-point scales tailored to each category. All sheets also recorded contextual information (date, type of intervention, observer, intervention coordinator, animal co-handler, time, and location). Data were collected in real time during each session by the two psychologists serving as intervention coordinators. Both psychologists were present at each session, and the assessments were jointly discussed and agreed upon, thereby ensuring consistency across observations.

To assess animal welfare during the cow-assisted interventions, we employed an observation sheet (Observation Sheet 5: Animal Welfare) developed in alignment with the Welfare Quality^®^ protocol for cattle [46]. In particular, the areas of evaluation were adapted from the criterion “Appropriate behaviour” and included: stress signs, inter-specific sociability, intra-specific sociability, and tolerance to handling. Table 4 presents the correspondence between our evaluation areas and the relevant Welfare Quality^®^ measures, ensuring consistency with established ethological frameworks.

Scores were expressed on a 1–5 scale (1 = minimum, 5 = maximum) for each indicator, and data were collected in real time twice a month during the sessions by the Veterinarian specialized in AAI. All the data were first collected via Google Form, but due to limited internet connectivity at the farm, paper-based sheets were subsequently used.

The five observation sheets have been reported in File S1.

All monitoring instruments, together with the informed consent form, were reviewed and approved by the Bioethics Committee of the University of Perugia.

### 2.5. Cost Analysis

The cost analysis was based on direct and indirect expenses associated with the implementation of AAIs. Direct costs included staff time, animal-related expenses, and user transportation, while indirect costs covered overhead such as administration, utilities, and insurance. Costs were estimated per 15-session cycle (45–60 min per session), with each item calculated on the basis of actual resource use and standard unit costs (Table 5).

The economic sustainability of cattle-assisted activities for the cooperative was estimated on the basis of the willingness of private clients or public health services to pay a price covering the costs of the activity plus a 10% margin to account for business risk. The financial sustainability of the investment was then assessed, both at the private scale (the Cooperative) and the public scale (regional funding), using the Net Present Value (NPV) method.

## 3. Results

The following section presents the empirical findings of the cow-assisted therapy program, resulting from the systematic monitoring of both beneficiaries and bovines. Results are organized into five main domains: psychological, emotional, relational, and nutritional outcomes for the three types of beneficiaries; animal welfare indicators; and an economic analysis of intervention costs. Data are reported through descriptive summaries and structured tables, providing a comprehensive overview of the observed changes and economic resource implications, while leaving their interpretation to the subsequent Discussion.

### 3.1. From Monitoring to Evidence: Main Findings

As required by the Italian guidelines on AAIs and in line with the study objectives, both participants and cows were systematically monitored during each session. The intervention coordinator was responsible for monitoring the participants, while the animal handler was responsible for monitoring the cow. At each session, both the coordinator and the handler carefully assessed and facilitated the well-being of all actors involved.

Beyond the collection of quantitative data, participants’ feedback was collected both through spontaneous expressions noted during the sessions and through a structured questionnaire administered at the end of the study. Qualitative observations made by the team revealed that many beneficiaries expressed genuine enjoyment in attending the weekly sessions at Forme dell’Anima. Whether due to a change in environment, the contact with nature, the interaction with the animals, or the activities themselves, participants frequently expressed appreciation for the program.

The quantitative observations were monitored with the five observational sheets discussed in Section 2. The assessments were defined as shown in Table 6. For each area, a maximum attainable score was established on the basis of the scoring criteria adopted and the number of items included. For example, within the category Emotional Expressions, five specific observations were considered: shows happiness, expresses disgust, expresses anger, shows fear, and avoidance. These five observations were grouped into two evaluation areas: positive emotional expressions (including the first observation) and negative emotional expressions (including the remaining four). For each observation, the evaluator could assign a score from 1 to 4 (1 = never, 2 = sometimes, 3 = almost always, 4 = always). Consequently, the maximum attainable score for the positive emotional expressions area was 4, while for the negative emotional expressions area it was 16. Then, for each area of evaluation, the average value of the first two months of observation (January–February 2024) and the last two months of observation (February–March 2025) was calculated and expressed in relation to the maximum value. This approach allowed us to highlight the progression between the initial and final phases of the project.

The monitoring results for the three types of beneficiaries are reported in Table 7, while Table 8 presents the monitoring results on animal welfare.

#### 3.1.1. Outcomes for Adolescents Removed from Families

Table 7 gives the results of the observational evaluation conducted with eight adolescents from the Bethel Community, who participated in a cycle of thirty sessions in the cow-assisted therapy project. In the psychological domain (anxiety, phobia, depressed mood, obsessive behaviors), a slight improvement was observed, with mean scores decreasing from 5.0 to 4.5 on a scale of 16 (31% to 28%), suggesting a modest attenuation of symptoms over time. Positive self-esteem, although showing a small decline (from 9.8 to 8.5 on a scale of 12), remained high relative to the maximum threshold (81% to 71%), indicating that participants continued to maintain a generally good self-perception. In parallel, negative self-esteem decreased slightly (from 2.8 to 2.5 on a scale of 8), consistent with a general improvement in self-perception.

In the emotional domain, positive emotional expressions (e.g., joy, satisfaction) increased significantly, from 3.0 to 3.8 on a scale of 4 (75% to 94%), reflecting greater positive effect. Conversely, negative emotional expressions (disgust, anger, fear, avoidance) decreased from 6.5 to 5.0 on a scale of 16 (41% to 31%), indicating an overall improvement in the emotional profile.

In the relational domain, positive relationships with peers improved (from 6.3 to 6.8 on a scale of 8, 78% to 84%), while negative relationships with peers decreased from 3.0 to 2.5 (38% to 31%), suggesting increased participation and social openness.

Findings regarding the interaction with the animal were particularly significant. Positive interaction (visual and tactile contact, play) remained high, with scores slightly decreasing from 13.0 to 12.2 on a scale of 16 (81% to 77%), confirming stable engagement with the animals. Negative interaction (discomfort or rejection of contact) remained low, shifting from 1.0 to 1.3 on a scale of 4 (25% to 31%), and thus stayed below the critical range, here defined as 50% or more of the maximum attainable score. Lastly, animal care behaviors (grooming, affectionate contact, feeding) showed improvement, increasing from 14.2 to 14.9 on a scale of 16 (approximately 89% to 93%). In this context, affectionate contact was defined as participants showing interest in the animal’s condition (e.g., observing it closely, asking about its well-being), while grooming activities included brushing, feeding, and stroking. These findings indicate greater responsibility toward and interest in the animal.

**Table 7:** Outcomes for adolescents removed from families, young adults with mental health problems, and individuals with eating disorders.

Areas of Evaluation/Beneficiaries (Participants)		Adolescents Removed from Their Families		Young Adults with Mental Health Problems		Individuals with Eating Disorders	
Number of Participants and Number of AAA Sessions		8 Adolescents-30 AAA		12 Young Adults-55 AAA		14 Individuals-65 AAA	
		Mean Score		Mean Score		Mean Score	
	Maximum Score	January–February 2024	February–March 2025	January–February 2024	February–March 2025	January–February 2024	February–March 2025
Psychological symptomatology (anxiety, phobia, depressed mood, obsessive behaviors). 4 observations	16.00	5.00	4.50	6.40	5.50	12.00	8.80
	100%	31%	28%	40%	34%	75%	55%
Positive self-esteem (ability, confidence, active participation). 3 observations	12.00	9.75	8.49	7.44	7.59	6.00	7.44
	100%	81%	71%	62%	63%	50%	62%
Negative self-esteem (insecurity, passivity). 2 observations	8.00	2.75	2.50	3.72	3.30	4.80	3.92
	100%	34%	31%	47%	41%	60%	49%
Positive emotional expressions (contentment). 1 observation	4.00	3.00	3.75	2.90	3.00	1.70	1.70
	100%	75%	94%	73%	75%	43%	43%
Negative emotional expressions (disgust, anger, fear, avoidance). 4 observations	16.00	6.50	5.00	5.72	4.60	6.80	6.80
	100%	41%	31%	36%	29%	43%	43%
Positive relationship with peers (active participation, interaction). 2 observations	8.00	6.25	6.75	5.90	6.40	4.60	5.60
	100%	78%	84%	74%	80%	58%	70%
Negative relationship with peers (withdrawal, lack of communication). 2 observations	8.00	3.00	2.50	3.00	2.50	4.26	3.26
	100%	38%	31%	38%	31%	53%	41%
Positive interaction with the animal (visual/tactile contact, play). 4 observations	16.00	13.00	12.24	11.00	13.60	9.92	11.32
	100%	81%	77%	69%	85%	62%	71%
Negative interaction with the animal (discomfort, refusal of contact). 1 observation	4.00	1.00	1.25	1.30	1.20	2.00	1.86
	100%	25%	31%	33%	30%	50%	47%
Animal care (grooming, affectionate contact, feeding). 4 observations	16.00	14.20	14.00	12.60	13.90	9.10	10.70
	100%	89%	88%	79%	87%	57%	67%
Attitudes toward milk and dairy products (sugars, fats, proteins, taste appreciation, frequency of consumption, increase in variety). 6 observations.	24.00			12.48	17.04	12.48	17.04
	100%			52%	71%	52%	71%

Note: Scores were coded as follows: 1 = never; 2 = occasionally; 3 = almost always; 4 = always.

#### 3.1.2. Outcomes for Young Adults with Mental Health Disorders

Table 7 also presents the results of the observational evaluation conducted with seven users of the “Si può fare” day-care center and five residents of the “Casa Avigliano” family-home. Participants took part in a cycle of fifty-five sessions within the cow-assisted therapy project.

In the psychological domain, a slight improvement was observed, with mean scores decreasing from 6.4 to 5.5 on a scale of 16 (40% to 34%), suggesting a modest attenuation of anxiety, depression, and obsessive behaviors. Positive self-esteem increased from 7.4 to 7.6 on a scale of 12 (62% to 63%), indicating a stable perception of self as capable and active. At the same time, negative self-esteem declined from 3.7 to 3.3 on a scale of 8 (47% to 41%), reflecting a reduction in feelings of insecurity and passivity.

In the emotional domain, positive emotional expressions (e.g., contentment) increased slightly, from 2.9 to 3.0 on a scale of 4 (73% to 75%), whereas negative emotions (e.g., anger, fear, avoidance) showed a more marked reduction, from 5.7 to 4.6 on a scale of 16 (36% to 29%), highlighting an overall improvement in emotional well-being.

In the relational domain, positive relationships with peers improved from 5.9 to 6.4 on a scale of 8 (74% to 80%), while negative relationships decreased from 3.0 to 2.5 on a scale of 8 (38% to 31%), suggesting greater social participation and reduced difficulties in interpersonal interactions.

The most significant finding concerns the interaction with the animal. Positive interaction (visual/tactile contact, play) increased substantially from 11.0 to 13.6 on a scale of 16 (69% to 85%), confirming the participants’ strong engagement with the animal. Negative interaction remained very low, with a slight reduction from 1.3 to 1.2 on a scale of 4 (33% to 30%), indicating minimal signs of discomfort or avoidance. Lastly, animal care behaviors (grooming, petting, feeding, and showing interest) improved markedly, from 12.6 to 13.9 on a scale of 16 (79% to 87%), pointing to increased responsibility and affection toward the animal.

#### 3.1.3. Outcomes for Individuals with Eating Disorders

Table 7 also summarizes the observational results obtained for two groups of participants from the “Città Giardino” Eating Disorder (ED) Community: the first group included 10 participants while the second group consisted of 4 participants for a total of 65 sessions. The evaluation domains were consistent with those used in other sheets of the cow-assisted therapy project, with the addition of a section specifically focused on attitudes toward the consumption of milk and dairy products, considered especially important in adolescents with eating disorders [47,48].

In the psychological domain, a clear improvement was observed, with scores decreasing from 12.0 to 8.8 on a scale of 16 (75% to 55%), indicating a marked reduction in anxiety, depressed mood, and obsessive behaviors. Positive self-esteem increased from 6.0 to 7.4 on a scale of 12 (50% to 62%), while negative self-esteem decreased slightly from 4.8 to 3.9 on a scale of 8 (60% to 49%), suggesting an overall improvement in self-perception.

In the emotional domain, positive emotional expressions remained stable at relatively modest levels (1.7 to 1.7 on a scale of 4; 43%), while negative emotions (disgust, anger, fear, avoidance) also remained unchanged at 6.8 on a scale of 16 (43%). This stability suggests that in this subgroup, emotional development may require longer or more targeted support.

In the relational domain, positive interactions with peers increased from 4.6 to 5.6 on a scale of 8 (58% to 70%), while negative relationships decreased from 4.3 to 3.3 on a scale of 8 (53% to 41%), indicating greater openness to group participation and improved social skills.

Regarding the interaction with the animal, positive interaction increased substantially from 9.9 to 11.4 on a scale of 16 (62% to 71%), while negative interaction decreased slightly from 2.0 to 1.9 on a scale of 4. Animal care behaviors (grooming, petting, feeding) showed a more notable improvement, from 9.1 to 10.7 on a scale of 16 (57% to 67%), reflecting a greater willingness to care for another living being, a potentially therapeutic element in ED treatment pathways.

Lastly, a particularly significant finding concerns attitudes toward milk and dairy products, an area specifically targeted for this population. The mean score increased from 12.5 to 17.0 on a scale of 24 (52% to 71%), highlighting a positive change in terms of variety, frequency, and openness to foods often avoided in EDs. This improvement may be interpreted as one of the most significant indirect effects of animal-mediated interaction, as the experiential and relational setting appears to facilitate a renegotiation of the relationship with dairy products.

#### 3.1.4. Cows and Animal Welfare

Alongside the monitoring of participant outcomes, the cow-assisted therapy project also included a structured assessment of animal welfare, focusing on the four cows involved in the activities (Table 8). Stress was primarily assessed through behavioral indicators observed during the AAI sessions, including repeated head shaking, leg movements such as walking in place, signs of agitation, and stepping back or withdrawing from the person involved in the activity. Stress signs remained constant at 1.8 on a scale of 5 (35% of the maximum threshold) from the beginning to the end of the project. This suggests a stable but contained level of stress, warranting further exploration through possible adaptations in animal engagement strategies. Importantly, the score did not increase, indicating no decline in welfare.

Inter-specific sociability was assessed through observable affiliative behaviors shown when people approached the paddock, including moving toward them (even toward strangers), sniffing their hands, licking their hands, and generally seeking close proximity. Inter-specific sociability showed a slight decrease from 12.8 to 12.0 on a scale of 15 (85% to 80%). Despite this reduction, values remained high, confirming a good predisposition to interspecific interaction, which is crucial for the effectiveness of Animal-Assisted Interventions (AAIs).

Intra-specific sociability was assessed through behaviors within the herd, including the absence of aggressive interactions, acceptance of other cows in close proximity, absence of social isolation, and tolerance in allowing all members of the herd to access resources such as feed and water, albeit according to the natural hierarchy of the group. Intra-specific sociability declined slightly from 5.0 to 4.0 on a scale of 5 (100% to 80%). Although still high, this change suggests a possible variation in group dynamics that warrants further environmental and management observation.

Tolerance to handling was assessed by observing the cows’ behavioral reactions when handled by the familiar handler, the veterinarian, or unfamiliar people. Indicators included the absence of startle responses, fear-related behaviors, or aggressive reactions during common management activities. Tolerance to handling also showed a slight decrease, from 17.0 to 16.0 on a scale of 20 (85% to 80%). Tolerance nonetheless remained high, confirming positive behavioral resilience despite the minor decline.

**Table 8:** Outcomes of monitoring Animal Welfare.

Areas of Evaluation on Animal Welfare	Maximum Score	Mean Score January–February 2024	Mean Score February–March 2025	Mean Score from January–February 2024 to February–March 2025
Number of Cows and Number of AAA Sessions		4 Cows-3 AAA	4 Cows-3 AAA	4 Cows-12 AAA
Stress signs. 1 observation	5.00	1.75	1.75	1.80
	100%	35%	35%	36%
Inter-specific sociability (with handler, staff, strangers including participants). 3 observations	15.00	12.75	12.00	12.90
	100%	85%	80%	86%
Intra-specific sociability (with the herd). 1 observation	5.00	5.00	4.00	4.25
	100%	100%	80%	85%
Tolerance to handling (by handler, staff, veterinarian, and inappropriate handling by strangers). 4 observations	20.00	17.00	16.00	17.00
	100%	85%	80%	85%

Note: Scores were expressed on a scale from 1 to 5, where 1 = minimum and 5 = maximum.

### 3.2. Cost Analysis

#### 3.2.1. Cost Analysis of Animal-Assisted Activities (AAA) and Animal-Assisted Education (AAE) Conducted with Cows

Table 9 shows the estimated costs of AAA and AAE conducted with cows, calculated for a cycle of 15 sessions and expressed also as the average cost per session. The analysis considers both human and animal-related resources, as well as indirect and logistical expenses, providing a comprehensive picture of the financial requirements of such interventions.

The main cost items are project design, including fees for both the veterinary expert (€200) and the project coordinator (€200). Among recurring costs, the animal handler, intervention coordinator, and accompanying staff represented the most significant expenditure (€75.75/session). In the case of AAA, the presence of the intervention coordinator is not required by national guidelines, while the presence of an accompanying operator is not systematically required; therefore, their costs were not included.

The most significant cost components are related to professional human resources. The animal handler, the intervention coordinator, and the accompanying operator each account for €25.25 per session, while the veterinary expert and project manager contribute a further €13.33 each. Together, these roles represent the largest proportion of the total costs, underlining the professional expertise required to ensure both educational value and participant safety.

Animal-related costs are relatively modest. This highlights that, although animal care is indispensable, the overall economic burden of AAA and AAE is primarily determined by human resources rather than animal upkeep.

Daily maintenance and training of the cow were estimated at €2.50/session, with an additional €150 allocated for healthcare expenses. These additional health costs are required to ensure that the cow employed in cow-assisted interventions complies with the hygienic and sanitary standards established by the national guidelines on AAI. The most significant item concerns the periodic Polymerase Chain Reaction (PCR) testing of hair samples for dermatophytosis (€52 every two months), which represents a critical preventive measure against zoonotic transmission. In the event of a positive result, a confirmatory sequencing analysis (€20) is required, constituting an occasional but necessary additional expense. The fecal analysis by coprological sedimentation (€8 every six months) ensures the detection of intestinal parasites, thereby further reducing potential health risks for both animals and beneficiaries. Lastly, the mandatory veterinary health certification (€70) provides formal assurance of the animal’s fitness to participate in assisted interventions, in compliance with veterinary public health standards. Given the timing of the analyses, additional veterinary expenses are incurred on average every 30 sessions (€5.0/session).

Adding together personnel and animal costs, the subtotal reaches €59.42 per session for AAA and €109.92 per session for AAE. When overheads (14%) are included, the figure rises to €67.74 and €125.31, respectively. Importantly, the calculation also incorporates transportation costs for beneficiary and operator (estimated at €6.50 per session, based on 10 km at 0.65 €/km), which are often underestimated in program planning but can significantly affect feasibility in rural or decentralized contexts.

In total, the overall cost per session amounts to €74.24 for AAA, corresponding to 1113.53 € for 15 sessions, and to €131.81 for EAA, corresponding to €1977.08 for 15 sessions.

#### 3.2.2. Cost Analysis of Animal-Assisted Therapy (AAT) Conducted with Cows

In the case of AAT activities, the hourly cost of a session is obtained by adding the hourly cost of an AAE session (€131.81) to the cost of monitoring and final evaluation of individual beneficiaries and animals, estimated at €24.93 (Table 10). All these costs refer to specialized staff. The table gives the breakdown of monitoring and evaluation costs for a cycle of 15 AAT sessions with 10 users. On the animal side, costs include in-presence monitoring, record-keeping, and a final evaluation of the cattle. On the user side, costs cover individualized project planning, user monitoring, and final evaluation. Additional components include setting and data collection, transportation costs, and the professional time of the veterinarian, project coordinator, animal handler, and intervention coordinator.

The largest shares are attributable to the project coordinator (€765) and the veterinarian expert (€665), together accounting for more than 75% of the total. Lower but still significant contributions come from the intervention coordinator (€252.50) and the animal handler (€187.50). Transportation expenses are marginal, at about €65 per professional involved. The total cost of monitoring and evaluation amounts to €1870 for 15 sessions with 10 users, corresponding to an average of €24.93 per user per session.

Therefore, based on the assumptions made, the overall cost for a single AAT session amounts to €156.74.

## 4. Discussion

### 4.1. Beneficiaries’ Reactions and Human–Animal Dynamics

Although cattle are considered “domestic animals,” having been domesticated for over 10,000 years [29,30], many participants reported never having approached or touched a cow. Even the project coordinator noted that she herself had never regarded cattle as “domestic” in the everyday sense of the word. Beneficiaries within each group showed diverse attitudes and responses toward the cows.

Some participants expressed fear or discomfort. For example, one adolescent and one adult woman appeared visibly tense in the presence of the animal. Other participants responded with immediate curiosity and engagement. Several took numerous photographs of the cows, often posing with them, suggesting enthusiasm and a positive attitude toward the interaction.

Ambivalent reactions were also observed, particularly among participants with family backgrounds in livestock farming or butchery. These individuals appeared to have complex and emotionally charged associations with cows, resulting in mixed feelings. Ambivalent reactions were identified through participants’ own statements. For instance, one boy reported that he felt comfortable with the cows but did not wish to continue the activities since he already had cows on his family farm. Similarly, one girl explained that she enjoyed being with the cows but considered such activities incompatible with the work in her family’s livestock business.

Participants’ emotional states directly influenced the animal’s behavior. In one case, a visibly frightened participant entered the stall to groom the cow. Their tenseness was evident, reacting abruptly to minor animal movements, while the cow showed alert behavior, as indicated by repeated head shaking. Immediately afterwards, another participant entered with a calm and confident approach. During grooming, the cow showed clear signs of relaxation—lowering its head, half-closing its eyes, and loosening its muscles.

This contrast highlighted the significant role of nonverbal communication and emotional state in shaping animal behavior during human–animal interactions.

Our results indicate that participants’ emotional states directly influenced the cattle’s behavior, confirming that animals are able to perceive and respond to human emotions [49]. This finding aligns with previous studies highlighting the central role of the human–animal bond in AAIs [50]. Such reciprocal effects may also be explained by neurophysiological mechanisms, such as the release of oxytocin, which fosters calmness, trust, and social bonding during human–animal interactions [51].

Approaching, touching, and even leading a cow—with its imposing size and strength—is not something to be taken for granted, especially for individuals experiencing a significant degree of insecurity or fear. However, surprising transformations were observed over the course of the intervention. One particularly significant case involved a boy who, at the beginning of the project, openly declared his fear of dogs and animals in general. Despite this, he consistently engaged in all proposed activities and ultimately completed the program by confidently and unhesitatingly leading the cow with a rope. Similarly, a girl who was initially reluctant to make physical contact with the cows gradually found her own respectful way of interacting, and eventually she was able to guide the cow calmly.

The scientific literature supports the idea that working with large animals can positively affect self-esteem [38,52]. Taking a small dog by the leash is one thing, but leading a cow, with its bulk and presence, is an entirely different experience—more demanding, yet profoundly rewarding.

Overall, the data suggest a positive emotional and relational development of the adolescents, with a general reduction in problematic aspects. Interaction with the cow emerged as a central catalyst for the observed changes, consistent with evidence in the literature on the beneficial effects of AAIs in educational and rehabilitative settings [26,53,54].

The project appears effective in promoting emotional well-being, boosting self-esteem, and fostering social and affective interaction for adults with mental health issues. The cow once again emerges as a central catalyst for positive change and inclusion. The longer duration of the intervention for the family-home group may have contributed positively to some outcomes, suggesting a potential relationship between session intensity and effectiveness.

Moreover, the data confirm the effectiveness of the cow-assisted therapy program for eating disorders, not only in emotional and relational dimensions, but also in relation to specific nutritional and behavioral objectives. The synergy between animal-assisted activity and clinical observation appears especially promising in this area.

Overall, the findings confirm that animal welfare was continuously monitored, with generally high values and no evidence of decline.

The integration of a systematic assessment of animal welfare within an AAI project is a fundamental quality, both ethically and operationally, and strengthens the sustainability of the intervention. In particular, attention to interspecific relationships and tolerance to handling provide a guarantee of safety for both operators and beneficiaries, as well as serving as a key indicator of the quality of the human–animal relationship established.

Our findings are consistent with previous evidence on the beneficial effects of AAIs in educational and rehabilitative settings [55,56]. Emotional and relational improvements observed in adolescents, along with a reduction in problematic aspects, mirror results from meta-analyses and experimental studies demonstrating that AAIs foster self-esteem, social skills, and emotional well-being in young people [57,58]. Similarly, the positive changes and enhanced inclusion observed among young adults with mental health problems align with systematic reviews highlighting the potential of AAIs to promote psychosocial functioning in clinical populations [59]. The effectiveness of the cow-assisted program for individuals with eating disorders is also consistent with prior work suggesting that AAIs can support motivational, relational, and even nutritional goals in psychiatric disorders [60]. Our data also confirmed that animal welfare was protected throughout the intervention. This important aspect supports literature asserting that continuous monitoring of therapy animal well-being is an ethical and operational cornerstone of sustainable AAI practice [61].

Direct qualitative observations suggested that the safety and effectiveness of cow-assisted activities were strongly influenced by the quality of the relationship between the cows and their handlers. The animals consistently turned to the handler for reassurance in situations of uncertainty, which mainly arose from the behavior of some participants—for example, when they appeared nervous or fearful in front of the cows, or when they expressed emotions in an excessively exuberant way (as in the case of one participant with a mental health condition) underscoring the central role of relationship in the human–animal dyad. Similar observations have been reported in other AAIs, where the handler is recognized as a key mediator of safety and animal welfare. In particular, studies on dog- and horse-assisted interventions emphasize that the handler not only prevents accidents but also provides reassurance; when the handler remains calm and relaxed, the animal is more likely to perceive the situation as safe and to display correspondingly relaxed behavior, which also benefits participants [61,62,63]. These parallels suggest that, regardless of species, the handler’s expertise and bond with the animal are a critical factor for minimizing risks and ensuring a positive environment for the animal.

### 4.2. Economic Sustainability of AAIs Conducted with Cows

The cost analysis shows that the largest share of costs is not that of animal care (approximately €7.50/session, including feeding, training, and healthcare), but rather that of professional and human resources (veterinary expert, intervention coordinator, activity or project coordinator, animal handler). The relatively low direct costs for the animal confirm the centrality of human–animal interaction as a professionalized and structured activity, rather than as a simple extension of animal husbandry.

A cross-model comparison of the three animal-assisted intervention formats—AAA, AAE, and AAT—reveals substantial differences in cost structures, largely reflecting the varying degrees of professional involvement, personalization, and therapeutic intensity.

The comparative analysis showed that while AAA can be implemented at relatively lower cost (this reflects the lighter structure of AAA, which emphasizes recreational and relational benefits rather than formal therapeutic goals), AAE represents a more resource-intensive model. This is justified by its stronger educational focus, the need for structured pedagogical planning, and the involvement of a larger multidisciplinary team.

Monitoring and evaluation represent a substantial share of the total costs of AAT, mainly due to the involvement of highly specialized professionals. This points out the importance of professional expertise in ensuring the quality and safety of the interventions, but also raises the issue of economic sustainability if the service is to be replicated or scaled up. Overall, the analysis confirms that the financial sustainability of AAT depends greatly on qualified professional involvement, while direct animal-related costs remain modest. The structure demonstrates how AAT programs prioritize safety, personalization, and ethical standards, justifying the relatively higher costs compared to other forms of animal-assisted interventions. The therapeutic model represents the most resource-intensive approach. The higher costs are driven by individualized project planning, continuous user monitoring, and systematic animal welfare assessments. Professional expertise accounts for the greatest share of expenditures, particularly the project manager and veterinary expert, reflecting the clinical rigor and ethical standards required for AAT. While AAA is the most cost-efficient due to its lighter structure, AAE introduces educational complexity and logistical costs, and AAT is the most professionally demanding, requiring intensive monitoring and individualized care. The comparative analysis demonstrates a clear trade-off: as the intervention shifts from recreational (AAA), to educational (AAE) and lastly to therapeutic (AAT), the financial investment increases in parallel with the level of professionalization, personalization, and expected clinical outcomes.

In all three models, animal-related costs remained modest, confirming that the financial sustainability of cow-assisted interventions is primarily determined by human resources and professional expertise rather than direct animal upkeep.

Once the costs of the individual AAI activities were identified, the economic sustainability for the cooperative was defined by the willingness to pay, either by private individuals or by public health services, a price covering the costs of the activity plus a 10% margin to account for business risk. This would imply that a user should be willing to pay €74.51 for one hour of AAA, €144.99 for one hour of AAE, and €172.41 for one hour of AAT. These prices refer to activities conducted with a single user, although such interventions are often carried out with groups.

Based on its resources and acquired expertise, the cooperative is able to offer cow-assisted activities across all three types of AAIs. Assuming the availability of four cows providing one session per day for 150 days per year, the annual supply amounts to 600 h. If these hours are evenly distributed across the three types of activities, total revenues would reach €78,384 against costs of €71,258. These flows would generate an operating profit of around €7000, in addition to the profit from the cooperative’s other activities.

When assessing the sustainability of the €150,000 investment made under project 16.9 solely in terms of the economic flows achievable by the cooperative over the next five years, the results indicate unsustainability. Indeed, the estimated Net Present Value (NPV), calculated over a 7-year horizon (with the first two years devoted to the investment) and assuming a 2% cost of capital, yields a negative result of approximately –€115,000. The investment would have been sustainable for the cooperative only with a contribution of €26,000, equal to 18% of the total investment.

It is therefore important to assess the sustainability of the investment from the perspective of the Region of Umbria, which funded the project. Assuming that the experimentation and results attract the attention of national authorities in charge of drafting and revising AAI guidelines, the investment becomes fully sustainable if, in addition to the cooperative, at least four other farms could adopt the results and implement the activities in the same way. Under this scenario, the NPV would reach about €16,000, and the Internal Rate of Return (IRR) would be 11%. Hence, if made replicable through regulation, the results of the project—and in particular the cow-assisted intervention model developed—would allow the public expenditure on this project to be considered both effective and efficient.

Our findings indicate that the largest share of costs in cow-assisted interventions is linked to professional and human resources rather than to the direct expenses of animal care. This pattern is consistent with the wider literature on AAIs, which repeatedly shows that the financial sustainability of such programs is primarily determined by the level of professional involvement, clinical supervision, and systematic monitoring, while direct animal-related costs remain modest [64,65]. A cross-model comparison also supports previous evidence: AAA is generally more cost-efficient due to its lighter structure and recreational focus, whereas AAE requires greater pedagogical planning and resources, and AAT represents the most resource-intensive model, demanding continuous monitoring, individualized planning, and specialized staff [59,66]. These differences in cost structure mirror the increasing degree of professionalization, personalization, and therapeutic rigor, which justify the higher costs of AAT compared to AAA and AAE [67]. Overall, the results of our cost analysis align with the literature in showing that while animal welfare and direct animal management are important and integrated into all activities, it is the human component—expertise, supervision, and professional care—that drives both quality and economic sustainability in AAIs.

Our findings are consistent with previous studies that highlight the benefits of animal-assisted interventions in promoting emotional well-being, self-esteem, and social functioning [59,64,65,66,67]. However, this study represents an innovative contribution by focusing on cow-assisted interventions, a scarcely explored field within the AAI literature, and by systematically integrating both human and animal welfare assessments as key indicators of intervention quality and sustainability. Furthermore, this is the first time that the economic sustainability of cow-assisted AAIs has been addressed.

## 5. Conclusions

Introducing a new species into Animal-Assisted Interventions (AAIs) was not straightforward, as it meant taking a path that was both unknown and difficult to validate. The results, however, were significant. In light of the outcomes obtained through cow-assisted activities, the expert team proposes repeating the experience in the form of cow-assisted therapies, subject to approval by the Ministry of Health. To achieve this goal, three steps will be required: (i) the veterinary AAI expert submitting a report documenting the results in terms of safety and absence of incidents to the Ministry of Health; (ii) development of a project involving the same animals, already proven to be reliable and docile; (iii) approval of the project by the regional ethics committee and the regional service responsible for public hygiene and safety. It will also be beneficial to invite public decision-makers to visit the cooperative and to participate, together with beneficiaries, in cow-assisted activities, thus experiencing first-hand the type of human–animal relationship that these animals are able to foster.

Beyond the clinical and relational aspects, the long-term success of cow-assisted interventions requires effective governance. Social farming, which integrates agricultural activities with social, educational, or healthcare interventions, depends on coordinated action among public institutions, the third sector, farms, and beneficiaries. Governance can be structured locally to create networks, foster participation, and ensure sustainability.

For cow-assisted interventions, governance operates on three levels. At the project level, it relies on shared processes and practices among partners. At the institutional level, it requires recognition by Local Health Authorities and the inclusion of AAIs within the range of services offered, ideally as part of the Individualized Assistance Plan. Municipalities could also play a crucial role by supporting and financing AAI initiatives in collaboration with local farms. At the regulatory level, however, a major obstacle remains: Italy is currently the only country that restricts the species of animals permitted in AAIs. The promising results of cow-assisted interventions should therefore be taken into account in future revisions of the national guidelines.

The authors are aware that this pilot study has several limitations, including the small sample size and the absence of a control group, which restricts the generalizability of the findings. It should also be acknowledged that, within the evaluation framework, some areas were composed of a single observation while others included multiple items. This heterogeneity may represent a methodological limitation, as it could affect the balance and sensitivity of the scoring system. Future studies should aim to refine these instruments by standardizing the number of observations across evaluation areas.

Nonetheless, it provides initial evidence on the feasibility and potential benefits of cow-assisted interventions. Further research should include controlled trials with larger samples to validate these results, as well as comparative studies across different species (e.g., cows versus horses) to identify species-specific benefits, challenges, and welfare implications. Also, future research should pay closer attention to environmental or seasonal variables in order to better interpret variations in cow behavioral indicators. By combining scientific validation, ethical responsibility for the well-being and dignity of vulnerable individuals, ethical attention to animal welfare, and institutional support, cow-assisted interventions could evolve into a replicable and sustainable model within the broader framework of Animal-Assisted Interventions.

## Figures and Tables

**Table 1:** Sociodemographic characteristics of the beneficiaries.

Beneficiaries	No.	Age	Male	Female
Adolescents removed from their families	8	12–16	5	3
Young adults with mental health problems	12	18–30	8	4
Individuals with eating disorders	14	16–22	2	12

**Table 2:** Structure of the experimental cow therapy protocol.

Structured Sessions	Content and Activities
1. Introduction to the project and the human–animal bond	Presentation of the project, objectives, and activities; exploration of the human–animal relationship with focus on mutual care; sharing stories and experiences to establish an emotional connection.
2. Getting to know cattle and their behavior	Introduction to cattle ethology (herd dynamics, human interactions); guided observation of cows in their environment; first direct but protected contact.
3. Personal experiences and first close contact	Participants shared past experiences with animals; initial gestures of care through the fence to strengthen trust and confidence.
4. Cattle in art, history, and creativity	Exploration of cultural and symbolic representations of cattle; creation of artworks using natural farm materials; further care and interaction through a fence.
5. Continuous care and strengthening the bond	Practical interaction focused on feeding and grooming through a fence, reinforcing the relationship.
6. Milk and its products	Learning about nutritional properties of milk; observation and participation in cheesemaking, highlighting its role in rural tradition.
7. Feeding and observing	Preparation of vegetables and hay for the cows; observation of animal behavior, preferences, and reactions to foster empathy and mindful observation.
8. Sound experiences and music therapy	Entry into paddock with live music; observation of cattle responses; animal care activities with music to create a calming atmosphere.
9. Animal welfare and collaboration	Reflection on physical, mental, and relational aspects of animal welfare; problem-solving activity with cattle to encourage respectful collaboration.
10. Leading the cow	With handler support, participants guided a cow with a halter along a short path, practicing trust, respect, and cooperation.
11. Milking	Introduction to the milking process; observation of mechanical milking and possible practice of manual milking; symbolic conclusion with hand milking, carried out using a traditional technique.

**Table 3:** Monitoring tools used in the study.

Instrument	Objective	Target	Observer	When
Observation Sheet 1: EMOTIONAL EXPRESSIONS and RELATIONSHIP WITH OTHERS	Assess ability to recognize, express, and share emotions; evaluate quality of social interactions	All human participants (beneficiaries)	Two Psychologists (intervention coordinators)	During each session
Observation Sheet 2: INTERACTION WITH THE ANIMAL and ANIMAL CARE	Document quality, frequency, and modalities of contact and engagement with the cow	All human participants (beneficiaries)	Two Psychologists (intervention coordinators)	During each session
Observation Sheet 3: PSYCHOLOGICAL SYMPTOMATOLOGY AND SELF-ESTEEM	Evaluate changes in psychological symptomatology and self-confidence	All human participants (beneficiaries)	Two Psychologists (intervention coordinators)	During each session
Observation Sheet 4: ATTITUDES TOWARDS MILK AND DAIRY PRODUCTS	Monitor attitudes toward milk and dairy products, reduction in anxieties/resistances, and inclusion of new foods	Human participants with eating disorders (EDs) (beneficiaries)	Two Psychologists (intervention coordinators)	During each session
Observation Sheet 5: ANIMAL WELFARE	Ensure continuous monitoring of the cow’s health and comfort	Cow involved in the intervention	Veterinarian specialized in AAI	Twice a month

Note: In all instruments, the term beneficiaries refers exclusively to the human participants.

**Table 4:** Correspondence between Observation Sheet 5 on Animal Welfare and Welfare Quality^®^ criteria for cattle.

Observation Sheet 5—Area of Evaluation	Indicators Used in This Study	Correspondence with Welfare Quality^®^ (2009) Criteria
Stress signs	Head shaking, ear and tail movement, stepping back	Appropriate behaviour → “Good human–animal relationship” (avoidance distance, flight reactions, agitation indicators)
Inter-specific sociability	Licking, sniffing, following handlers/staff, accepting food and cuddles from participants	Appropriate behaviour → “Good human–animal relationship” (positive contact tests, willingness to approach humans)
Intra-specific sociability	Licking, sniffing, accepting closeness with other bovines	Appropriate behaviour → “Expression of social behaviours” (affiliative interactions, social grooming, tolerance of proximity)
Tolerance to handling	Acceptance of haltering, milking, feeding, cleaning, dressing, veterinary care, and tolerance to inappropriate handling by participants	Good housing and Appropriate behaviour → “Absence of pain induced by management procedures” (ease of handling, tolerance to common farm/veterinary practices)

**Table 5:** Cost components of AAIs (15-session cycle, 45–60 min each).

Cost Item	Description
Program design and planning	Involves the veterinary AAI expert and the project coordinator for designing the 15-session cycle
Operational staff	Cost of the animal handler
Beneficiary support staff	Cost of the operator accompanying the beneficiary (if required)
Animal-related costs	Daily maintenance, training, and additional veterinary care to ensure animal welfare
General overhead	Administrative expenses, utilities, and insurance
Beneficiary transportation	Costs for transferring beneficiaries to and from the facility

**Table 6:** Methods for deriving values from observational data.

Observation Sheet	No. Observation	Areas of Evaluation	Maximum Attainable Score
Observation Sheet 3: PSYCHOLOGICAL SYMPTOMATOLOGY AND SELF-ESTEEM	4	**Psychological symptomatology**	16
		Anxiety	
		Phobias	
		Depressed mood	
		Obsessive behaviors	
	3	**Positive self-esteem**	12
		Easily follows proposed actions	
		Feels rewarded and capable	
		Is active and proactive	
	2	**Negative self-esteem**	8
		Feels inadequate and insecure	
		Is passive and indifferent	
Observation Sheet 1: EMOTIONAL EXPRESSIONS and RELATIONSHIP WITH OTHERS	1	**Positive emotional expressions**	4
		Shows happiness	
	4	**Negative emotional expressions**	16
		Expresses disgust	
		Expresses anger	
		Shows fear	
		Avoidance	
	2	**Positive relationship with peers**	8
		Participates actively	
		Interact with others	
	2	**Negative relationship with peers**	8
		Participates only when asked	
		Does not speak to anyone	
Observation Sheet 2: INTERACTION WITH THE ANIMAL and ANIMAL CARE	4	**Positive interaction with the animal**	16
		Maintains eye contact	
		Seeks tactile contact	
		Accepts the animal’s approach	
		Plays with it	
	1	**Negative interaction with the animal**	4
		Is annoyed by contact with the animal	
	4	**Animal care**	16
		Brushes it	
		Feeds it	
		Strokes/pets it	
		Is interested in knowing how the animal is doing	
Observation Sheet 4: ATTITUDES TOWARDS MILK AND DAIRY PRODUCTS	6	**Attitudes toward milk and dairy products**	24
		Nutrients: sugars	
		Nutrients: fats	
		Nutrients: proteins	
		Appreciation of taste	
		Frequency of consumption	
		Increase in variety	
Observation Sheet 5: ANIMAL WELFARE	1	**Stress sign**	5
		Head shaking, ear and tail movement and stepping back	
	3	**Inter-specific sociability**	15
		Licking, sniffing, following the handlers	
		Licking, sniffing, following the observers	
		Licking, sniffing, accepting food and cuddles from participants	
	1	**Intra-specific sociability**	5
		Licking, sniffing, and accepting the closeness with other bovines	
	4	**Tolerance to handling**	20
		Accepting to be guided with a halter, to be milked, fed, cleaned, and dressed by the handlers	
		Accepting to be milked, fed, cleaned, and dressed by the observer	
		Tolerance to inappropriate handling by participants	
		Accepting dressings and injections by the veterinarian	

**Table 9:** Cost analysis of Animal-Assisted Activities (AAA) and Animal-Assisted Education (AAE) conducted with cows (Values in Euros).

	Animal-Assisted Activities (AAA)		Animal-Assisted Education (AAE)	
Cost Items	Aggregate Cost for a 15-Session Cycle	Unit Cost per Session	Aggregate Cost for a 15-Session Cycle	Unit Cost per Session
Veterinary specialized in AAIs	200.00	13.33	200.00	13.33
Activity coordinator (AAA), Project coordinator (EAA)	200.00	13.33	200.00	13.33
Animal handler		25.25		25.25
Intervention coordinator	unexpected cost			25.25
Accompanying operator	unexpected cost			25.25
Cow, daily cost including training		2.50		2.50
Cow, additional health costs	75.00	5.00	75.00	5.00
Total cost for staff and cow		59.42		109.92
Overheads (14% of total costs)		8.32		15.39
Overall costs		67.74		125.31
Estimated transportation expenses for beneficiary and operator, calculated as 10 km at €0.65 per km		6.50		6.50
Overall costs, including transportation expenses	1113.53	74.24	1977.08	131.81

**Table 10:** Cost breakdown for monitoring and evaluation in Animal-Assisted Therapy (AAT) (values in euros).

Cost Items	Veterinary Specialized in AAIs	Activity/Project Coordinator	Animal Handler	Intervention Coordinator	Overall
Animal monitoring in presence (1.5 h × 4 times)	300.00				
Animal monitoring, completion of records (4 h)	200.00				
Final evaluation of animals (2 h)	100.00				
Individualized beneficiares projects (5 h)		250.00			
Beneficiaries monitoring (5 h)		250.00			
Final evaluation of beneficiaries (4 h)		200.00			
Setting and data collection (0.5 h per session)			187.50	187.50	
Transportation costs (25 km × €0.65/km)	65.00	65.00		65.00	
Aggregate design and monitoring costs for 15 sessions with 10 beneficiaries	665.00	765.00	187.50	252.50	1870.00
Unit design and monitoring cost per session per beneficiaries	8.87	10.20	2.50	3.37	24.93

## Data Availability

The dataset generated during the current study will be made available from the corresponding author on reasonable request.

## Supplementary Materials

The following supporting information can be downloaded at: https://www.mdpi.com/article/10.3390/ani15202957/s1. File S1: Observation Sheets of Cow-Assisted Therapy.

## Abbreviations

AAIs	Animal-Assisted Interventions
AAA	Animal-Assisted Activity
AAE	Animal-Assisted Education
AAT	Animal-Assisted Therapy
UN	United Nations
EU	European Union
IAHAIO	International Association of Human–Animal Interaction Organizations
NPV	Net Present Value
ED	Eating Disorder
PCR	Polymerase Chain Reaction
IRR	Internal Rate on Return
